# Ultrasmall nanostructured drug based pH-sensitive liposome for effective treatment of drug-resistant tumor

**DOI:** 10.1186/s12951-019-0550-7

**Published:** 2019-11-29

**Authors:** Yanyan Li, Yongxia Zhai, Wei Liu, Kaixiang Zhang, Junjie Liu, Jinjin Shi, Zhenzhong Zhang

**Affiliations:** 1 The Fifth Affiliated Hospital of Zhengzhou University, Kangfu Road, Zhengzhou, 450052 China; 2 School of Pharmaceutical Sciences, Zhengzhou University, Zhengzhou, China; 3 Collaborative Innovation Center of New Drug Research and Safety Evaluation, Zhengzhou, Henan China; 4 Key Laboratory of Targeting Therapy and Diagnosis for Critical Diseases, Zhengzhou, Henan China

**Keywords:** Reversing drug resistance, Drug efflux, Nuclear-transport, Nanostructured drug, Programmatic release

## Abstract

**Background:**

Cancer cells always develop ways to resist and evade chemotherapy. To overcome this obstacle, herein, we introduce a programmatic release drug delivery system that imparts avoiding drug efflux and nuclear transport in synchrony via a simple nanostructured drug strategy.

**Results:**

The programmatic liposome-based nanostructured drugs (LNSD) contained two modules: doxorubicin (DOX) loaded into tetrahedral DNA (TD, ~ 10 nm) to form small nanostructured DOX, and the nanostructured DOX was encapsulated into the pH-sensitive liposomes. In the in vitro and in vivo studies, LNSD shows multiple benefits for drug resistance tumor treatment: (1) not only enhanced the cellular DOX uptake, but also maintained DOX concentration in an optimum level in resistant tumor cells via nanostructure induced anti-efflux effect; (2) small nanostructured DOX efficiently entered into cell nuclear via size depended nuclear-transport for enhanced treatment; (3) improved the pharmacokinetics and biodistribution via reducing DOX leakage during circulation.

**Conclusions:**

The system developed in this study has the potential to provide new therapies for drug-resistant tumor.

## Background

The emergence of multiple-drug resistance (MDR) is remaining a main obstacle for successful treatment of cancer [1]. Cancer cells often develop drug resistance and stop responding to chemotherapeutics after repeated sessions of chemotherapy [2]. MDR can be induced by various mechanisms, including decreased drug uptake, increased drug efflux, activation of detoxifying systems, activated of DNA repair mechanisms and evasion of drug-induced apoptosis [3–5]. In particular, MDR is typically mediated by the overexpression of a membrane transporter, P-glycoprotein (P-gp), actively increases the efflux of drugs from cancer cells [6, 7]. The efflux drugs reduced the therapeutic effect and cancer cells often develop drug resistance and stop responding to chemotherapeutics [8].

To reverse MDR, great effort has been devoted to developing specific drug delivery systems (DDS) [9, 10]. In the past 10 years, nanoscaled drug delivery systems such as liposome [11], solid lipid nanoparticles (SLN) [12], polymer micelles [13], mesoporous silica [14], carbon nanomaterial [15], and gold nanomaterial [16]. etc., which increased tumor selectivity and reduced toxicity have been receiving a lot of attentions. More importantly, the developed DDS could bring more drugs into the resistant tumor cells, therefore, significantly improved the antitumor efficacy [17]. Besides improving drug uptake via DDS, how to maintain the drug concentration in an optimum level in the resistant tumor cells is another main challenge for reversing MDR [18, 19]. To overcome the obstacle, the strategy of the super small nanoparticle was used. Firstly, compared with the free small molecule drugs, the small nanoparticle could not be efflux from drug resistant tumor cells, thus maintain the drug concentration [20]; Secondly, the super small nanoparticle could efficiently enter into the cell nuclear, and this is important for treatment. For example, Liang, etc. have developed a series of gold nanoparticles with different sizes, and they found the small gold nanoparticles could efficiently enter into the cell nuclear [21]. Therefore, in this study, the small nanoparticle was used for reversing MDR in tumor cells.

Recently, DNA nanotechnology has been widely investigated in different biomedical fields [22–24]. Tetrahedral DNA nanostructures (TDNs) have attracted a great deal of attention in biomedical fields due to the biological nature of DNA and convenient synthesis [25]. More importantly, TDNs have a super small size (~ 10 nm), and have a high drug loading efficacy [26]. TDNs have been considered a promising drug delivery system for cancer treatment [25–27]. In this study, TDNs were used as the secondary drug delivery vehicle.

The in vivo kinetic behavior of TDNs is a key point for tumor treatment [28], and a pharmacokinetic standpoint as nanoparticles less than 10 nm have been reported to be cleared by kidney, while larger nanoparticles have been reported to preferentially home into tumors through leaky tumor neovasculature as a result of the enhanced permeability and retention (EPR) effect [29, 30]. Liposomes are widely accepted as targeted delivery systems for antitumor drugs as demonstrated by the commercialization success of a number drug molecules [31]. pH-sensitive liposomes, usually PEGylated (pPSL), have been investigated to refine conventional liposomes in effective targeted extra- and intra-cellular delivery of anticancer drugs [32]. To improve the in vivo kinetic behavior of TDNs, pH-sensitive liposomes were used as the senior drug delivery vehicles.

**Fig. 1:**
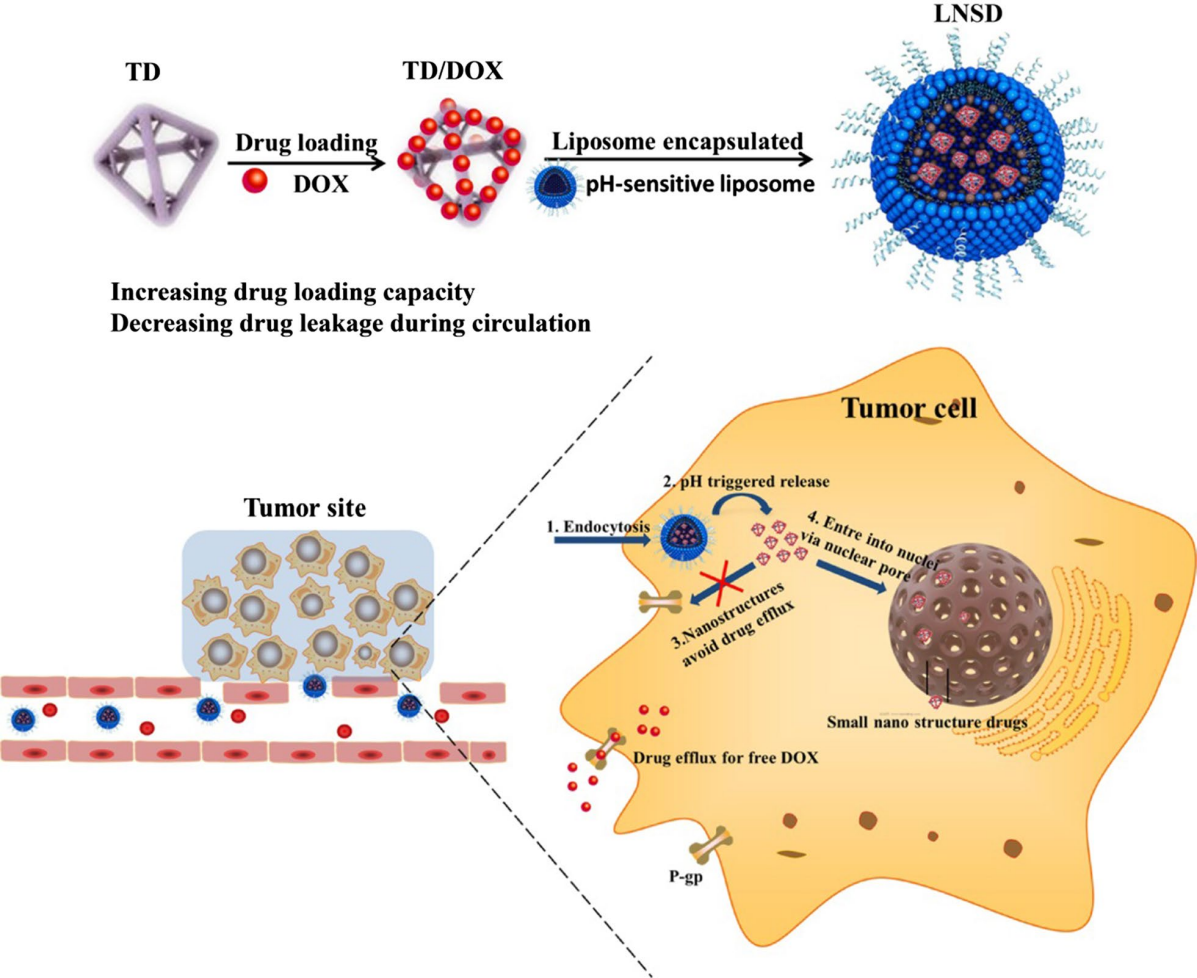
The preparation, anti-efflux and cell nuclear-transport effects of LNSD

In current study, we rationally designed a pH-sensitive liposome-based nanostructured DOX (LNSD) for drug resistance tumor treatment. As schemed in Fig. 1, DOX was loaded into TD to form the small nanostructured DOX (TD/DOX), and then the nanostructured DOX was encapsulated into the pH-sensitive liposomes to form LNSD. The prepared LNSD has multiple benefits for drug resistance tumor treatment: (1) not only enhanced the cellular DOX uptake, but also maintained DOX concentration in an optimum level in resistant tumor cells; (2) the small nanostructured DOX could enter into the cell nuclear for enhanced treatment; 3) accumulated in tumor site via EPR effect of liposomes. The enhanced antitumor efficacy and reversing DOX resistant effect of LNSD were investigated using MCF-7/ADR cells and DOX-resistant breast tumor models.

## Results

### Synthesis and characterization of LNSD

**Fig. 2:**
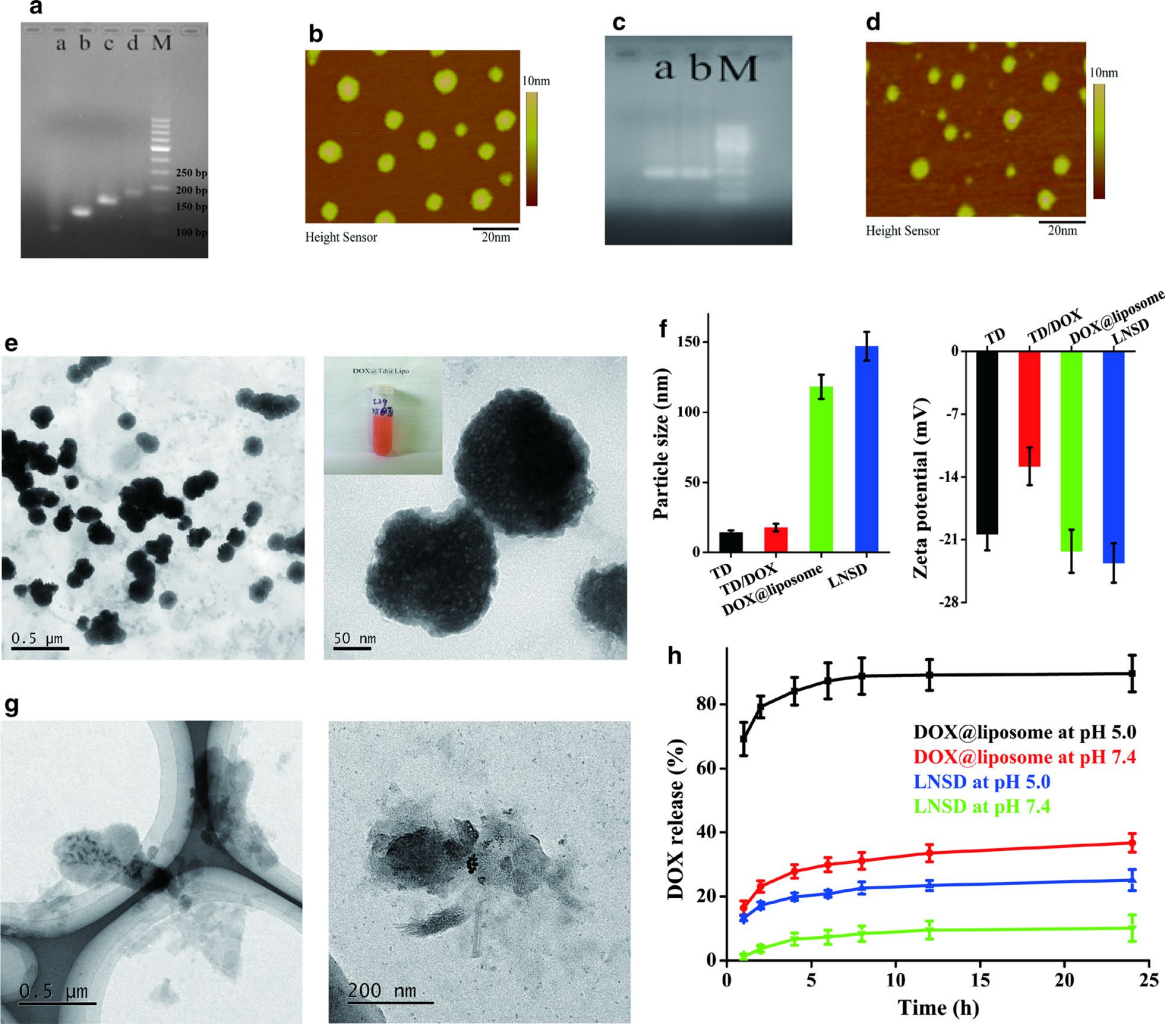
Synthesis and characterization of LNSDs. **a** Electrophoretic analysis of TD, a: S1 alone, b: S1 + S2, c: S1 + S2 + S3, d: S1 + S2 + S3 + S4 (TD), M: marker. **b** AFM image of TD. **c** Electrophoretic analysis of TD and TD/DOX, a: TD, b: TD/DOX, M: marker. **d** AFM image of TD/DOX. **e** TEM images of LNSD, insert: photo of LNSD nanosuspension. **f** Size and zeta potential of TD, TD/DOX, DOX@liposome and LNSD (n = 3). **g** TEM images of LNSD at pH 5.0 buffer for 8 h. **h** DOX release from DOX@liposome or LNSD at pH 5.0 and pH 7.4, respectively (n = 3)

Tetrahedral DNA nanostructure was assembled with four 55-mer strands (Table 1) prepared with a high-yield, single-step synthesis originally reported by Turberfield et al. [33]. The successful preparation of TD was confirmed by the results of electrophoresis (Fig. 2a). Along with the self-assembly of S1 (55 bases), S1(55 bases) + S2(55 bases), S1(55 bases) + S2(55 bases) + S3(55 bases) and S1(55 bases) + S2(55 bases) + S3(55 bases) + S4(55 bases), the migration speed was gradually decline, and TD was composed of S1 + S2 + S3 + S4 (total 220 bases) (Fig. 2a). AFM results showed the size of TD was ~ 10 nm (Fig. 2b), and the average size of TD was 14.3 ± 1.6 nm confirmed by DLS (Fig. 2f). DOX loading was achieved by incubating TD with DOX for 12 h at room temperature. After DOX loading, the zeta potential of TD showed a significant decrease (from − 20.4 to − 12.8 mV, Fig. 2f). While after DOX loading, the results of AFM, electrophoresis and DLS showed no significant difference compared to TD (Fig. 2c, d and f), showing DOX loading did not influence the structure of TD. Finally, pH-sensitive liposomes were used to encapsulate the DOX loaded TD (TD/DOX) to obtain the liposome-based nanostructured DOX (LNSD). The pH-sensitive liposomes were prepared using a thin-film hydration method. The particle size distributions of liposome, TD@liposome, DOX@liposome and LNSD were shown in Additional file 1, and the average particle size of LNSD was ~ 147 nm (Fig. 2f). TEM images showed LNSD had a uniform size and a ball-like structure (Fig. 2e). The DOX encapsulation efficiency (EE) of LNSD was calculated to 29.6%. Interestingly, the DOX encapsulation efficiency of DOX@liposome was 16.7%, which was much lower than that of LNSD, suggesting that the strategy of nanostructured DOX could significantly increase the EE of liposomes (Additional file 2).

**Table 1:** DNA oligonucleotides used in TD

ssDNA	sequence (5′–3′)	Base number
S1	ACATTCCTAAGTCTGAAACATTACAGCTTGCTACA CGAGAAGAGCCGCCATAGTA	55
S2	TATCACCAGGCAGTTGACAGTGTAGCAAGCTGTAA TAGATGCGAGGGTCCAATAC	55
S3	TCAACTGCCTGGTGATAAAACGACACTACGTGGGA ATCTACTATGGCGGCTCTTC	55
S4	TTCAGACTTAGGAATGTGCTTCCCACGTAGTGTCG TTTGTATTGGACCCTCGCAT	55
FAM-S1	FAMACATTCCTAAGTCTGAAACATTACAGCTTGCT ACACGAGAAGAGCCGCCATAGTA	55

The pH-sensitivity of LNSD was investigated by intuitive observation of the morphological change of LNSD in pH 5.0 buffer. According to the results of TEM (Fig. 2g), when LNSD was incubated in pH 5.0 buffer for 8 h, the shell of LNSD obviously ruptured, showing the high pH-sensitive ability of LNSD. Furthermore, the DOX release from DOX@liposome and LNSD were shown in Fig. 2h, in the case of DOX@liposome at pH 7.4, ~ 36.7% of DOX was released. While when the pH was declined to 5.0, the release of DOX significantly increased to ~ 89.6%, indicating the pH sensitive ability of the prepared liposomes. On the other hand, in the case of LNSD at 5.0 group, only ~ 25.1% of DOX was released after 24 h, much lower than that of DOX@liposome at pH 5.0 group (~ 89.6%), demonstrating most of DOX was not released from TD (Fig. 2h). More importantly, compared with DOX@liposome (free DOX strategy), LNSD (nanostructured DOX strategy) significantly decreased the unexpected DOX leakage (10.1% vs 36.7% after incubation with pH 7.4 buffer for 24 h, Fig. 2h), and the results were also confirmed by the photos of DOX@liposome and LNSD after centrifugation (Additional file 3).

### Anti-efflux and cell nuclear distribution effects of LNSD in MCF-7/ADR cells

MCF-7/ADR cells were used as the model cells in the in vitro studies. Firstly, the biodistribution of TD@liposome was investigated, and the TD was labeled via FAM. The results were shown in Additional file 4. After incubation with FAM-TD@liposome for 4 h, a large amount of the green fluorescence (FAM) were observed in MCF-7/ADR cells, and most of the fluorescence were in the cytoplasm; While when the incubation time was prolonged to 12 h, a considerable part of green fluorescence were in the cell nuclear, indicating that TD could enter into the cell nuclear.

The intracellular stability of TD determined the distribution of the TD/DOX in the cells, a lot of previous studies had proved that TD could retain structural integrity in cells. [25, 30, 34]. Next, to investigate the intracellular stability of TD/DOX encapsulated in LNSD, FAM-TD/DOX@pH-sensitive liposome was first prepared, and after incubating with the MCF/ADR cells for 4 h, the results was shown in Additional file 5. According to the results, the green fluorescence of TD (FAM) and red fluorescence of DOX displayed well-overlapped, confirming that DOX was not released from DOX-loaded TD within cells, thus demonstrating the stability of DOX-loaded TD in MCF/ADR cells.

**Fig. 3:**
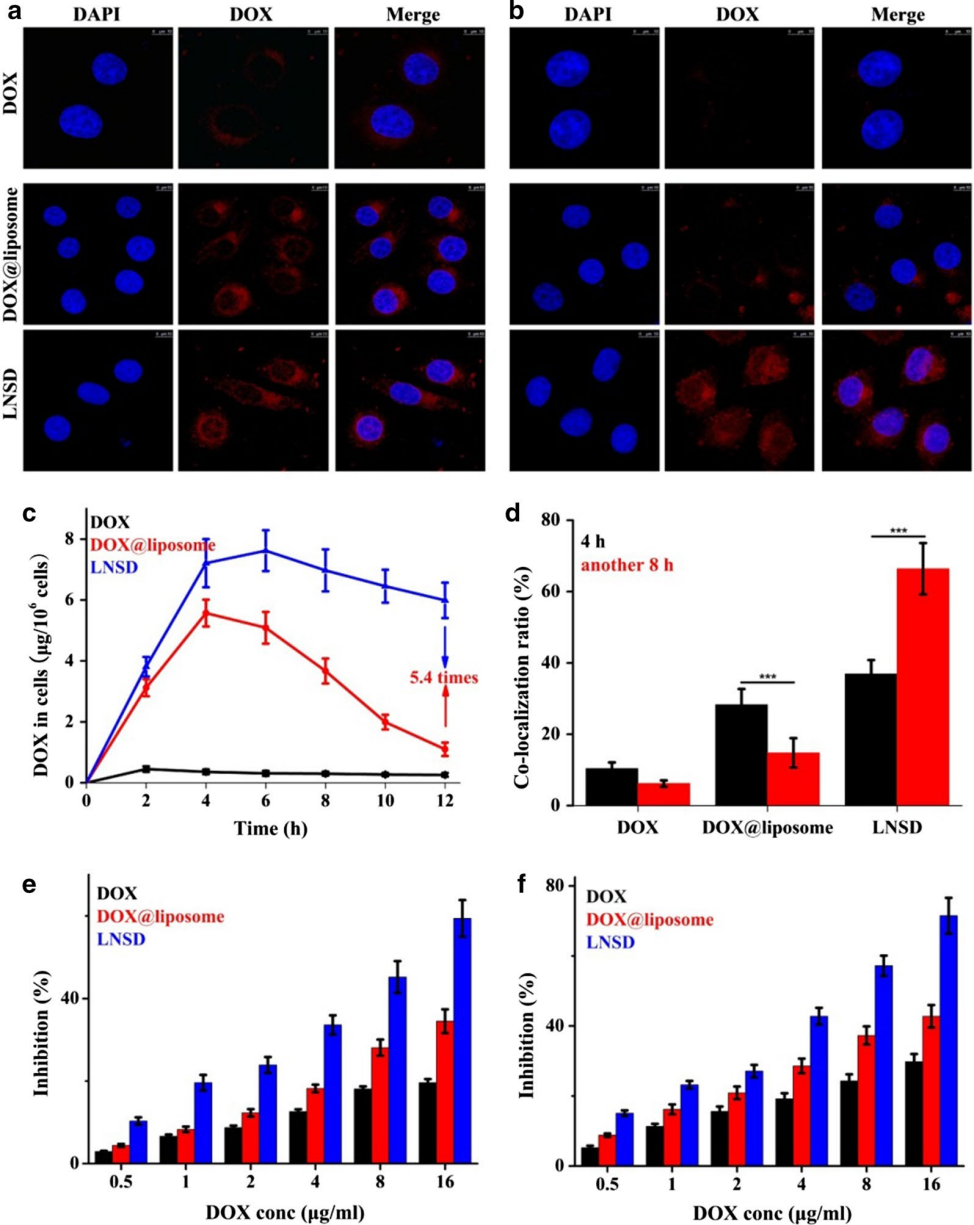
Anti-efflux, cell nuclear distribution and proliferation inhibiting effects of LNSD in MCF-7/ADR cells. **a** CLSM images of MCF-7/ADR cells treated with DOX, DOX@liposome and LNSD for 4 h, scale bar: 10 μm. **b** CLSM images of MCF-7/ADR cells treated with DOX, DOX@liposome and LNSD for another 8 h, the total incubation time was 12 h, scale bar: 10 μm. **c** Intracellular pharmacokinetics of DOX, DOX@liposome and LNSD (n = 4). **d** Co-localization ratio of DOX (red FLR) and cell nuclear (blue FLR) in DOX, DOX@liposome or LNSD treated MCF-7/ADR cells (n = 20). **e**, **f** The cell proliferation inhibition rates of DOX, DOX@liposome and LNSD with different DOX concentrations for 24 h and 48 h (n = 6). Data presented are means ± SD. ***p < 0.01

Next, we investigated the intracellular distribution of LNSD in MCF/ADR cells, and the results were shown in Fig. 3a and b. After incubation with DOX for 4 h, only weak red fluorescence (DOX) were found in MCF-7/ADR cells, while in the case of DOX@liposome and LNSD groups, more fluorescence were observed, indicating that LNSD or liposomes could carry DOX into the MCF-7/ADR cells. Interestingly, compared with DOX@liposome, more fluorescence was found in the cell nuclear (Fig. 3a). In the case of LNSD group, the co-localization ratio of DOX and DAPI (blue fluorescence) was significant higher than that of DOX@liposome (36.9% vs 28.3%, Fig. 3d). When the incubation time was prolonged to 12 h, almost no red fluorescence was detected in DOX group, indicating the efflux effect of MCF-7/ADR cells (Fig. 3b). The significant efflux effect was also found in the case of DOX@liposome group: compared with incubation for 4 h, the signals of red fluorescence in cells significantly decreased after another 8 h of incubation (Fig. 3b). However, in the case of LNSD group, and with the passage of incubation time, the red fluorescence of DOX did not show significant decrease (Fig. 3b).

The efflux effects in DOX, DOX@liposome and LNSD groups were also investigated via detecting the concentration of DOX in cells for different incubation times, and the results were shown in Fig. 3c. In the case of DOX group, the concentration of DOX in MCF-7/ADR cells was very low at every time point, while the concentration of DOX was much higher in DOX@liposome and LNSD groups. According to the results, after incubation for 4 h, the DOX concentration in cells decreased very fast in the case of DOX@liposome group, and after incubation for 12 h, the DOX concentration decreased to ~ 1.1 μg (Fig. 3c). On sharp contrast, the DOX concentration in cells decreased much slower in the case of LNSD group, and after incubation for 12 h, the DOX concentration was still much higher (~ 5.9 μg, Fig. 3c). The results showed the released DOX in cytoplasm could be discharged from the resistant cancer cells, while when DOX was encapsulated in nanoparticles (TD), the efflux effect of the resistant cancer cells was much weaker. More importantly, not like DOX and DOX@liposome groups, the co-localization ratios all showed significant decrease with the passage of incubation time, the co-localization ratio of LNSD showed a significant increase (from ~ 36.9% to ~ 66.4%, Fig. 3d). The above results showed not only the anti-efflux effect, but also the nuclear transport ability of the prepared LNSD.

The in vitro antitumor effect was highly correlated with the concentration of DOX in MCF-7/ADR cells, and the results were shown in Fig. 3e and f. As expect, the inhibition of LNSD was much higher than that of DOX or DOX@liposome after incubation for 24 or 48 h. When the cells were treated by LNSD (DOX concentration: 16 μg/mL) for 48 h, the inhibition was calculated to 71.5%, much higher than that of DOX (29.8%). More importantly, the inhibition of LNSD was also much higher than that of DOX@liposome (42.8%), indicating that the higher concentration of DOX in LNSD treated cells than that of DOX@liposome group. The toxicity of the blank carrier (TD@liposome) to MCF-7/ADR cells was also investigated, and the results were shown in Additional file 6, indicating that the blank carrier had a low toxicity to MCF-7/ADR cells.

### In vivo studies of LNSD

**Fig. 4:**
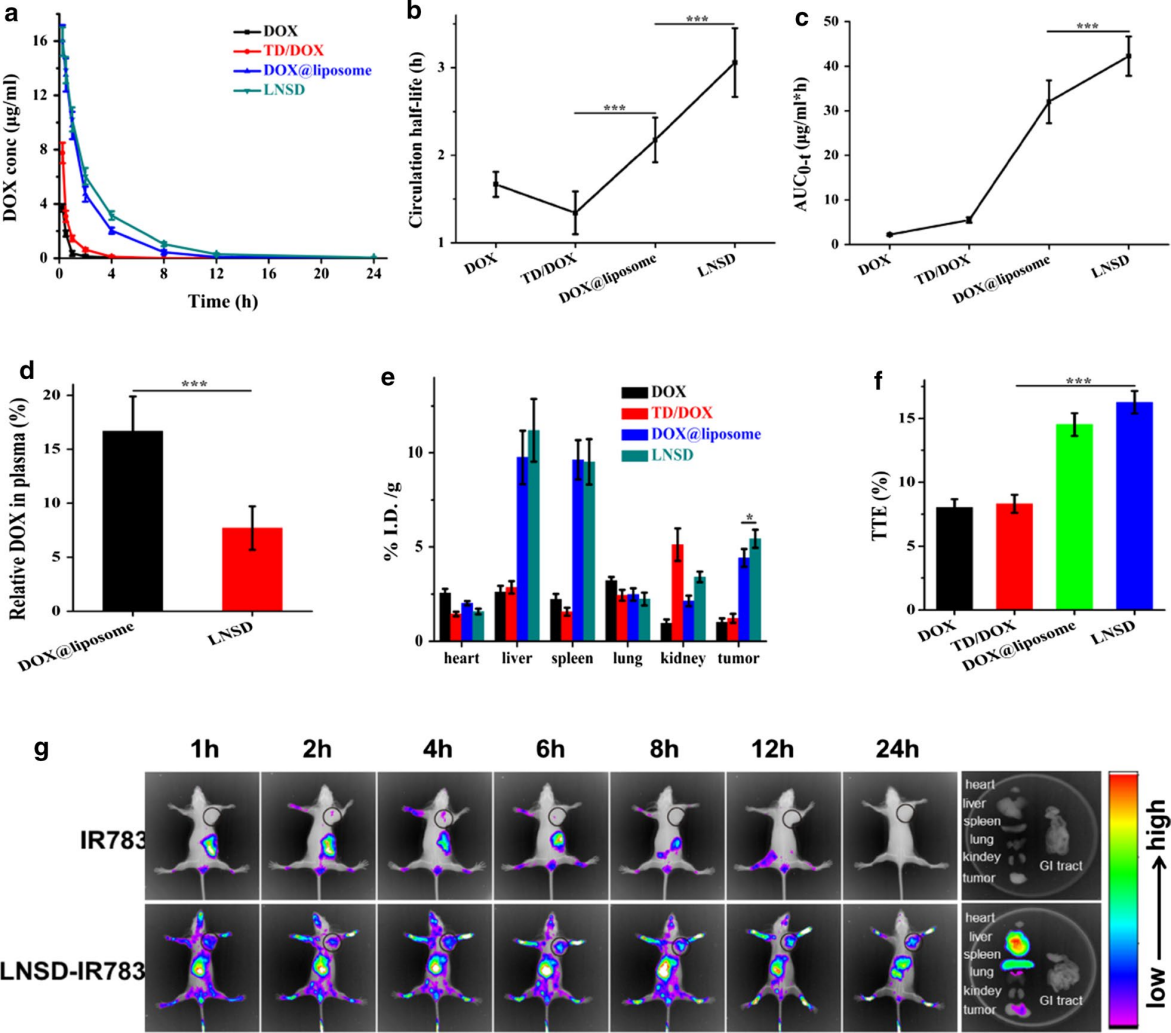
Pharmacokinetics and biodistribution. **a** Mean concentration of DOX in plasma after intravenous administration of DOX, TD/DOX, DOX@liposome and LNSD (n = 3, with the same DOX dosage of 5 mg/kg). **b** Circulation half-lives of DOX, TD/DOX, DOX@liposome and LNSD obtained by fitting the circulation profile data (n = 3). **c** AUC of DOX, TD/DOX, DOX@liposome and LNSD after injection (n = 3). **d** To determine the DOX leakage of DOX@liposome and LNSD, the drug-containing NPs were incubated with plasma for 4 h and the level of DOX was determined in the plasma (n = 3). **e** Biodistribution of DOX, TD/DOX, DOX@liposome and LNSD over a span of 24 h after injection (n = 3). **f** Tumor-targeting efficacy of DOX, TD/DOX, DOX@liposome and LNSD over a span of 24 h after injection (n = 3). **g** In vivo optical images of tumor-bearing mice at different time after injection with IR783 loaded LNSD. Data presented are means ± SD. ***p < 0.01

The pharmacokinetics of DOX, TD/DOX, DOX@liposome and LNSD were shown in Fig. 4a, showing the decrease of DOX in LNSD group was slower than that of DOX, TD/DOX or DOX@liposome after administration. The circulation half-life of LNSD was 3.059 h, much longer than that of DOX (1.667 h), TD/DOX (1.342 h) or DOX@liposome (2.176 h) (Fig. 4b). The area under the curve (AUC) of LNSD (42.267 μg/mL*h) was about twenty times greater than that of DOX (2.215 μg/mL*h) and about eight times greater than that of TD/DOX (5.499 μg/mL*h). The AUC of LNSD was also greater than that of DOX@liposome (42.267 vs 32.044 μg/mL*h, Fig. 4c). The results of pharmacokinetics indicated that compared with the other groups, LNSD significantly increased the blood circulation time and the bioavailability of DOX.

The leakage of DOX from the NPs could influence the pharmacokinetic behavior in vivo, therefore, the leakages of DOX from DOX@liposome and LNSD in plasma were investigated (Fig. 4d). After incubation with plasma for 4 h, in the case of DOX@liposome group, the relative DOX in plasma was 16.7%, however, the DOX leakage in LNSD group significantly decreased (7.7%), indicating that LNSD with the nanostructured DOX strategy could significantly increase the blood circulation time of DOX via reducing the leakage of DOX in vivo.

Next, we investigated biodistribution of DOX in various organs (heart, liver, spleen, lung, kidney and tumor), and there were significant differences for the biodistribution of DOX in DOX, TD/DOX, DOX@liposome and LNSD treated tumor-bearing mice (Fig. 4e). After injection for 24 h, the level DOX in LNSD treated group was 5.433, about 5.4, 4.5 and 1.2-times higher than that of DOX (1.011), TD/DOX (1.220) and DOX@liposome (4.423). The tumor-targeting efficacy (TTE) of LNSD, DOX, TD/DOX or DOX@liposome was 16.3%, 8.02%, 8.32% or 14.52%, respectively (Fig. 4f), indicating that LNSD had the best tumor-targeting ability. Besides that, the tumor-targeting ability of LNSD was also confirmed by a real-time imaging system (Fig. 4g). According to the results, after injection of free IR783 and LNSD-IR783, whole body distribution were observed in tumor-bearing mice, a significant tumor site accumulation were observed in the case of LNSD-IR783, and the FLR signal increased with the extend of time. At the same time, no obvious fluorescence in the tumor site was observed after free IR783 treated group, because the unstable IR783 could be rapidly excreted from body. It is worth noting that the fluorescence intensity of LNSD in liver and spleen are also high, which may attribute to higher blood perfusion in these organs.

**Fig. 5:**
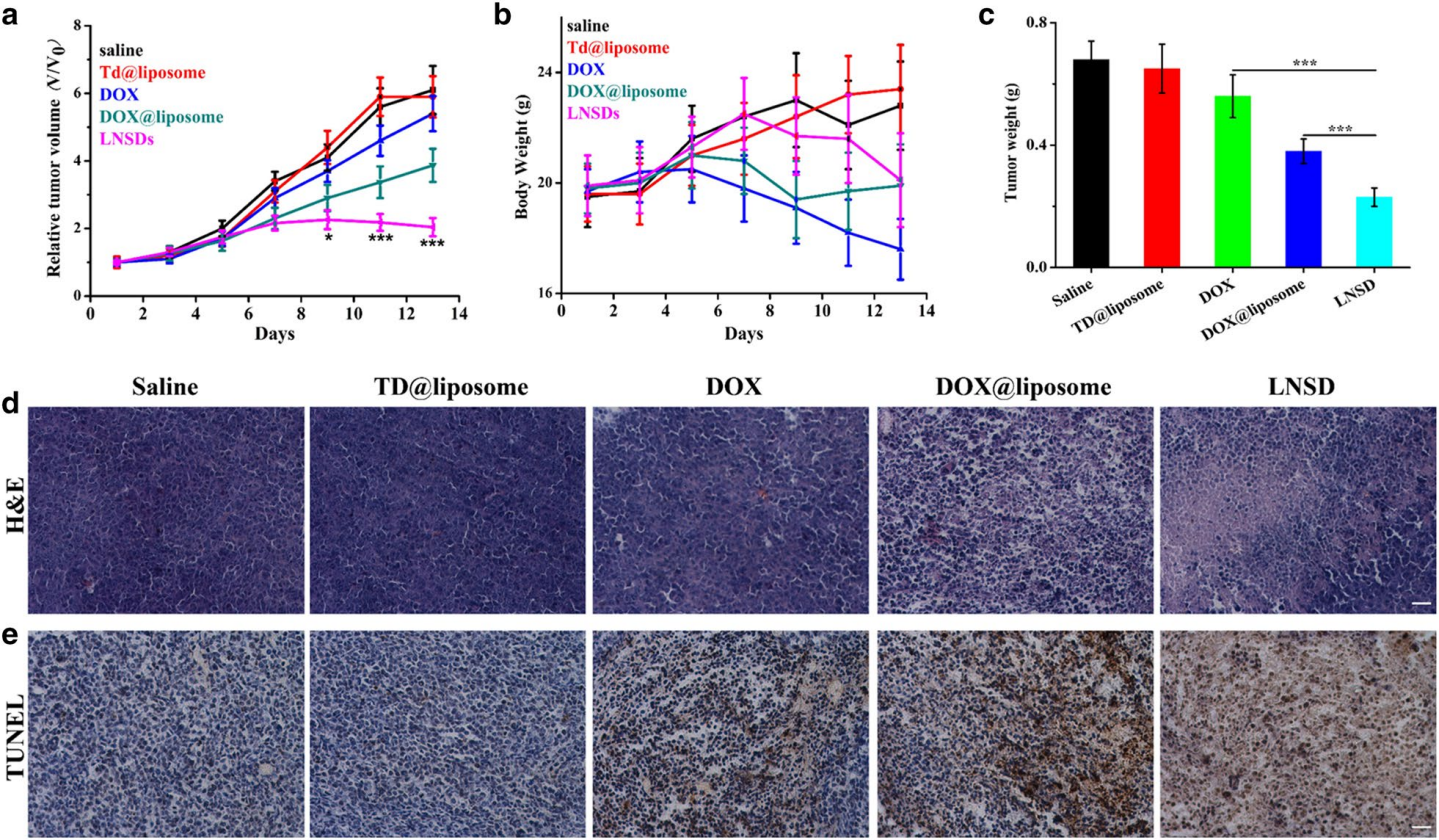
Antitumor activities of LNSD in MCF-7/ADR tumor-bearing mice. **a** Tumor volume changes of mice (n = 6) injected with LNSD or saline. **b** Body weight monitoring of the mice received different treatments. **c** Tumor weights of mice received the treatments measured on day 14. **d** Representative H&E staining images of tumor tissue received different treatments, scale bar: 100 μm. **e** Representative TUNEL staining images of tumor tissue received different treatments, scale bar: 100 μm. Data presented are means ± SD. *p < 0.05, ***p < 0.01

The MCF-7/ADR tumor-bearing mice were receiving different treatments for 15 days, and the results were monitored in terms of tumor volume change (Fig. 5a). In the case of control and TD@liposome (blank drug carrier) group, the MCF-7/ADR tumor-bearing mice were closely monitored for the continuous growth of tumor, which grew ~ 6 times larger on day 15 than it was initially, indicating that the blank drug carrier (TD@liposome) did not show any significant influence to the tumor growth. No significant difference of the relative tumor volume was shown between control group and DOX treated group, indicating the drug resistance property of MCF-7/ADR. Compared with DOX group (~ 5.40), the relative tumor volume of DOX@liposome significantly decreased to ~ 3.87, indicating the DOX delivery ability of the as-prepared liposome. More important, compared with DOX group (~ 5.40) and DOX@liposome group (~ 3.87), the relative tumor volume in LNSD showed a significantly decrease (2.04), demonstrating the tumor-targeting DOX delivery and reversing resistance abilities of LNSD. The tumor weights of different groups were shown in Fig. 5c, and the results were consistent with the relative tumor volume.

The weight loss of the tumor-bearing mice during different treatments was shown in Fig. 5b, according to the results, in the cases of control group and the blank drug carrier (TD@liposome) group, the tumor-bearing mice both gained the weight. In the cases of DOX@liposome group and LNSDs group, no significant weight loss was observed compared to the control group. While, in the case of DOX group, after treatment for 15 days, the weight of tumor-bearing mice significantly decrease, indicating the potential toxicity of DOX.

The therapeutic efficacy was also evaluated by the histological tissue images through H&E and TUNEL staining. As shown in Fig. 5d, severe tumor tissue damage was clearly observed in the LNSD-treated tumor compared with tumors in other groups, especially in DOX@liposome group. The results of apoptosis in different treatments were also shown in Fig. 5e, according to the results, a large number of apoptotic cells (dyed brown) were clearly observed in the case of LNSD-treated tumor, and the apoptotic cells were much more than that of saline, blank carrier, DOX and DOX@liposome-treated tumors, indicating that the therapeutic efficacy of LNSD.

**Fig. 6:**
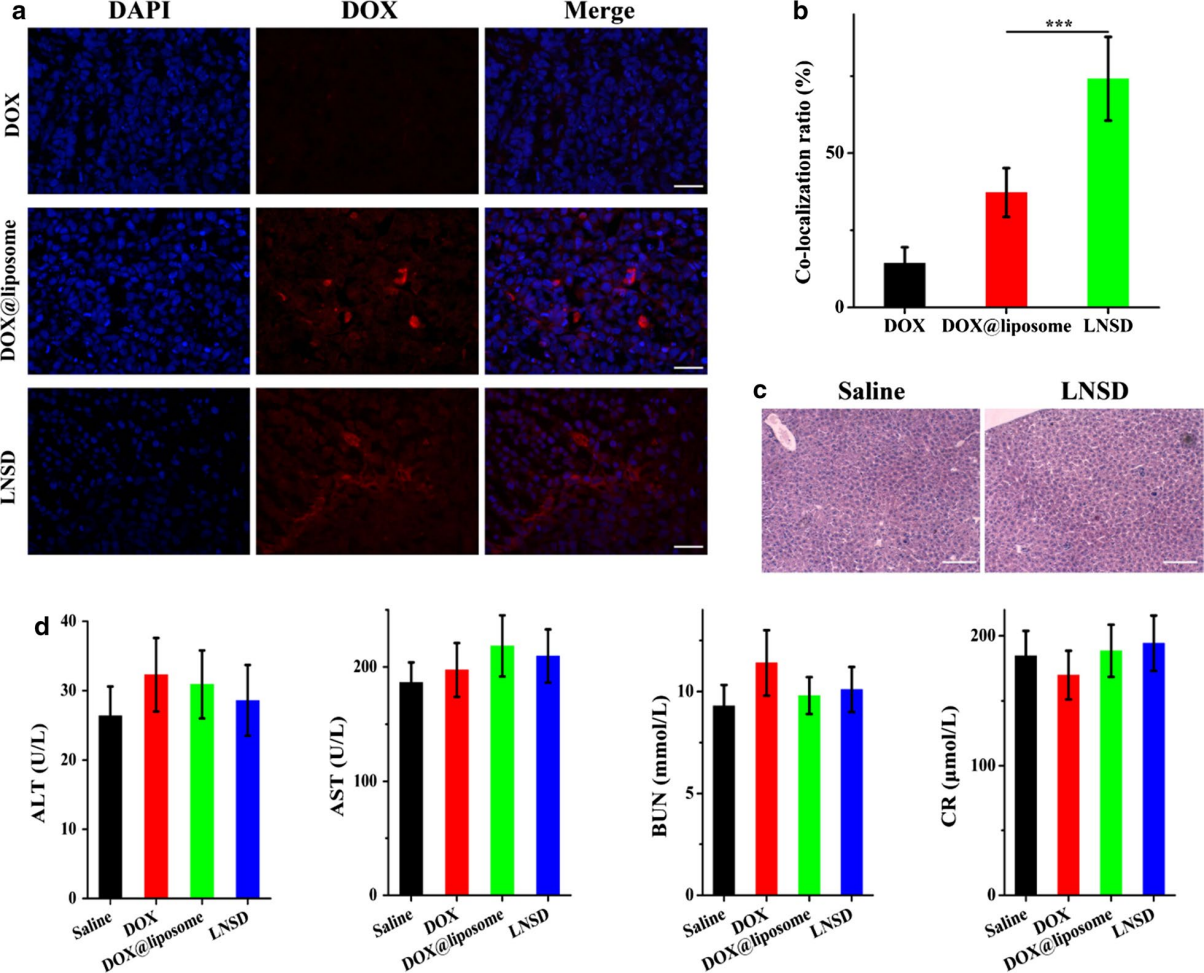
In vivo anti-efflux, cell nuclear distribution and biosafety studies. **a** CLSM images of tumor tissues harvested from DOX, DOX@liposome or LNSD treated MCF-7/ADR tumor-bearing mice at 24 h post injection (red FLR: DOX; blue FLR: DAPI), scale bar: 100 μm. **b** Co-localization ratio of DOX (red FLR) and cell nuclear (blue FLR) in tumor tissues harvested from DOX, DOX@liposome or LNSD treated MCF-7/ADR tumor-bearing mice (n = 20). **c** Representative H&E staining images of liver tissue received saline or LNSD treatments, scale bar: 200 μm. **d** ALT, AST, BUN and CR in serum were detected in different treatments on day 14 (n = 5). Data presented are means ± SD. *p < 0.05***p < 0.01

The cell nuclear-targeting ability of LNSD in tumor-bearing nude mice was investigated after injection of DOX, DOX@liposome and LNSD for 24 h. As shown in Fig. 6a, in the case of DOX group, only little DOX signal (red fluorescence) was observed, indicating the poor tumor-targeting of DOX. Compared with DOX-treated group, the DOX signals of DOX@liposome and LNSD showed significantly increase, demonstrating the tumor-targeting abilities of liposome-based drug delivery. More importantly, in the cases of DOX and DOX@liposome groups, the DOX signals were mainly in cytoplasm (Fig. 6a), and the overlap degree of DOX and the cell nuclear (DAPI, blue fluorescence) were ~ 14.3% and ~ 37.2%, respectively (Fig. 6b). However, in the case of LNSD group, the co-localization ratio of DOX and DAPI was increased to ~ 74.1% (Fig. 6b), indicating that most of DOX were in the cell nuclear, further demonstrating the cell nuclear-targeting ability of TD/DOX, and the results were consistent with the in vitro studies.

In vivo toxicity is a great concern in the development of nanomedicine, and according to the results of biodistribution, the nanomedicine were mainly in liver after injection, therefore, the toxicity of LNSD in liver was evaluated by H&E staining (Fig. 6c), and no significant toxicity was observed via the pathological section. Furthermore, alanine aminotransferase (ALT), aspartate aminotransferase (AST), blood urea nitrogen (BUN), and creatinine (CR) which are highly reflected the physiological state of liver and kidney were also investigated, and no significant difference between LNSD-treated mice and the saline-treated mice (Fig. 6d).

## Discussion

The unexpected release of drug during circulation always lead to the unexpected side effects and low therapeutic efficiency [35]. So an ideal drug delivery system should avoid the drug leaks as possible. In this study, DOX was not directly loaded into a liposome, instead, DOX was firstly loaded into a tetrahedral DNA nanostructure (TD) to obtained a nanostructured DOX, and then the DOX-loaded TD (nanostructured DOX) was enveloped via liposome. The DOX release from LNSD was much lower than that of DOX@liposome (DOX was directly loaded into the liposome) after 24 h, (~ 9.4% vs ~ 36.7%). This is beneficial to the pharmacokinetics and biodistribution of DOX, therefore, reduced the side effects and enhanced the delivery efficacy of DOX [36].

The emergence of drug resistance is a main obstacle for successful treatment of cancer [37]. Generally, typical drug resistance is mediated by the overexpression of a membrane transporter, P-glycoprotein (P-gp), actively increases the efflux of drugs from cancer cells [38]. In this study, DOX was loaded into TD to obtain a (TD/DOX), when LNSD entered into the MCF/ADR cells via endocytosis, the nanostructured TD/DOX was released into the cell cytoplasm, unlike free DOX, which is efflux via P-gp rapidly, the nanostructured DOX could not be efflux from MCF/ADR cells even after 12 h of incubation. The nanostructured TD/DOX significantly increased the intracellular accumulation of DOX in MCF-7/ADR cells and maintained a relative high DOX concentration in an optimum level.

Furthermore, the size of the nanostructured TD/DOX was ~ 15 nm, and the diameter of the nuclear pore is ~ 30 nm [39], so due to the small size of the nanostructured DOX complex, the DOX complex could enter into the cell nuclear via nuclear pore, making the nanostructured DOX have the cell nuclear-targeting ability, and this was also proved both in the in vitro and in vivo studies. The therapeutic target of DOX is the tumor cell nuclear, the cell nuclear-targeting ability is very important for DOX delivery. In general, LNSD showed not only the anti-efflux effect, but also the nuclear transport ability, and both the abilities are crucial for reversal of tumor resistance to DOX. This reversal of tumor resistance of LNSD effect was substantiated by: 1) significantly increase the DOX concentration in MCF-7/ADR cells in vitro and increase the DOX levels in the MCF-7/ADR tumor-bearing mice when compared to DOX and DOX@liposome (DOX was directly loaded into the liposome); 2) significantly improved the DOX distribution in the nuclear of MCF-7/ADR cells in vitro and in vivo compared to DOX and DOX@liposome; 3) significantly enhanced the antitumor efficiency in MCF-7/ADR cells in vitro and in MCF-7/ADR tumor-bearing mice in vivo compared to DOX and DOX@liposome.

## Conclusions

In total, a liposome-based nanostructured DOX (LNSD) was rational designed and investigated. In the in vitro and in vivo studies, LNSD could efficiently enter into the MCF-7/ADR cells and accumulated in the tumor tissue in MCF-7/ADR tumor-bearing mice. Furthermore, the DOX resistance of MCF-7/ADR cells could be reversed via the anti-efflux effect and the size-depended cell nuclear-targeting ability of the nanostructured DOX in LNSDs. Therefore, LNSD significantly improved the antitumor efficacy of DOX and the liposome-based DOX in drug-resistant breast tumor-bearing mice.

## Methods

### Materials

Doxorubicin (DOX, 20,170,511, purity >98%) was gotten from Beijing Yi-He Biotech Co. Ltd. All single-stranded DNAs were purchased from Takara Bio Co. (Dalian, China), and the specific sequences were displayed in Table 1. Phospholipids, 1,2-dioleoy-sn-glycero-3-phosphoethanolamine (DOPE), 1,2-dipalmitoyl-sn-glycero-3-phosphocholine (DPPC), 1,2-distearoyl-sn-glycero-3-phosphocholine (DSPC) and cholesteryl hemisuccinate (CHEMS) were purchased from Avanti Polar Lipids (Alabama, USA), and N-(carbonyl-methoxy-polyethylene-glycol-2000)-1,2-distearoyl-sn-glycero-3-phosphoethanolame (DSPE-mPEG2000) from Lipoid (Steinhausen, Switzerland). Cholesterol and calcein were obtained from Sigma-Aldrich Ltd (Auckland, New Zealand). PCR primer, loading Buffer, and Golden View was obtained from Beijing Ding Guo Chang Sheng Biotechnology Co. Ltd. Gel Extraction Kit, 2× Taq Master Mix, and 50× TAE was obtained from Beijing Com Win Biotech Co. Ltd. Sulforhodamine B (SRB), DMEM cell culture medium, penicillin, streptomycin, fetal bovine serum (FBS), and heparin sodium were bought from Gibco Invitrogen. DAPI, hematoxylin and eosin were supplied by Beyotime Biotechnology Co. Ltd. Other reagents were acquired from China National Medicine Corporation Ltd.

### Synthesis of LNSD

#### Synthesis of tetrahedral DNA nanostructure (TD)

TDs were synthesized as previously reported [33]. In brief, the single-stranded DNA S1, S2, S3 and S4 were mixed in buffer (10 mM Tris–HCl, 50 mM MgCl_2_.6H_2_O, pH 8.0) at the same concentrations. The mixture solution was next quickly heated to 95 °C for 5 min, and then the mixture solution was cooled down to 4 °C for 0.5 min. The FAM marked TDs (TD-FAM) were prepared under the same conditions using FAM-S1, S2, S3 and S4.

### Preparation of DOX-Loaded TD (TD/DOX)

DOX (500 μM) was added to the prepared TD solution (1 mM), and then the mixture was incubated for 12 h at room temperature with shaking (100 rpm) to make sure DOX was sufficiently intercalated into TD, after that, the mixture was centrifuged (10,000 rpm, 10 min) to remove unloaded DOX, and DOX-loaded TD (TD/DOX) was obtained.

### pH-sensitive liposome wrapping

The blank pH-sensitive liposome (liposome), DOX loaded pH-sensitive liposome (DOX@liposome) and LNSD were prepared using a thin-film hydration method. In detail, for the blank pH-sensitive liposome, DOPE, DSPC, CHEMS, cholesterol and DSPE-PEG2000 (molar ratio 4:2:2:2:0.3) were dissolved in chloroform and methanol mixture (3:1 v/v, 2 mL) and dried using a rotary evaporator under vacuum. After that, 1.5 mL of water was added, and the mixture (emulsion) was sonicated at room temperature for 30 min. For DOX@liposome and LNSD, with the same steps of dying, after that, 1.5 mL of DOX solution (500 μM) and 1.5 mL of TD/DOX solution was added, and the mixture (emulsion) was sonicated at room temperature for 30 min. The liposome suspension was then extruded through 0.2 μm pore sized polycarbonate membrane filters (Whatman, UK) using a 10 mL LIPEXTM Extruder (Northern Lipids Inc., Burnaby, Canada).

The loaded DOX in DOX@liposome or LNSD was determined by lipid emulsification method. In brief, 2 mL of methanol was added to 0.2 mL of DOX@liposome or LNSD, after sonicated at room temperature for 1 h, the samples were centrifuged (10,000 rpm, 10 min), and then the amount of the loaded DOX was determined by high performance liquid chromatography (HPLC, 1100 Agilent, USA) with the following conditions: an Eclipse XDB-C18 column (150 mm × 4.6 mm, 5.0 μm); mobile phase sodium acetate solution (0.02 mol/L)/acetonitrile 80: 20; column temperature 40 °C; fluorescence detector with the excitation and emission wavelengths set at 475 nm and 560 nm, respectively; flow rate 1.0 mL/min; and injection volume 20 μL. The DOX encapsulation efficiency (EE) was calculated using the following formula:

$$EE=\frac{\mathrm{W}}{\mathrm{W}_{0}}\times 100\%$$

W was the weight of DOX loaded into liposomes. W_0_ was the weight of DOX@liposome or LNSD.

### Characterization

DLS (Zetasizer Nano ZS-90, Malvern, UK), TEM (Tecnai G2 20, FEI) and AFM (SPM-9700, Shimadzu, Japan) were used for characterizing zeta potential, particle size and morphology of LNSD and TD, respectively. Polyacrylamide gel electrophoresis (PAGE, 8%) was used to verify TD and TD/DOX.

### DOX release profile

DOX@liposome (1 mL) and LNSD (1 mL) with the same DOX concentration (1 mg/mL) were sealed in the dialysis membranes (MW cutoff 12–14 KD, Spectrapor). The dialysis bags were incubated in 10 mL of PBS buffer (pH 7.4) and acetate buffer (pH 5.0), respectively. The released DOX was quantified by HPLC.

### In vitro studies using MCF-7/ADR cells

#### Cell culture

MCF-7/ADR cells (human breast cancer cell line, P-gp highly expression) were cultured in normal DMEM culture medium with 10% fetal bovine serum (FBS) and 1% penicillin/streptomycin in 5% CO_2_ and 95% air at 37 °C in a humidified incubator.

#### Cellular distributions of TD-FAM@liposome

MCF-7/ADR cells were seeded at 5 × 10^4^ cells per well in 6-well plates. When cells reached 70% confluence, they were treated with TD-FAM@liposome in the dark for 4 and 12 h. After staining with DAPI, the cells were washed with PBS for 3 times and imaged by a Confocal Microscopy (Zeiss, LSM 700, Germany).

#### Intracellular stability of TD/DOX

MCF-7/ADR cells were seeded at 5 × 10^4^ cells per well in 6-well plates. When cells reached 70% confluence, they were treated with TD-FAM/DOX@liposome in the dark for 4 h. After 4 h of incubation, the cells were washed with PBS for 3 times and imaged by a Confocal Microscopy (Zeiss, LSM 700, Germany).

#### Cellular internalization studies

MCF-7/ADR cells were seeded at 5 × 10^4^ cells per well in 6-well plates. When cells reached 70% confluence, they were treated with DOX, DOX@liposome and LNSD (with the same DOX concentration: 10 μg/mL) in the dark for 4 h. After staining with DAPI, the cells were washed with PBS for 3 times and imaged by a Confocal Microscopy. After imaging, the cells were incubated for another 8 h (the total incubation time was 12 h), and then the cells were imaged by a Confocal Microscopy again.

#### In vitro antitumor studies

MCF-7/ADR cells were plated in 96-well plates and then incubated for 24 h. After incubation, the cell medium was replaced with fresh culture medium containing DOX, DOX@liposome and LSND (with the same concentration of DOX) for 4 h, respectively, and then the medium was replaced with the fresh medium. After incubation for another 20 or 44 h (the total incubation time was 24 h or 48 h), standard SRB was carried out to determine the cell viabilities.

#### Detection of DOX in MCF-7/ADR cells

MCF-7/ADR cells were treated with DOX, DOX@liposome and LNSD (with the same DOX concentration 20 μg/mL). At the designated time points (2, 4, 6, 8, 10 and 12 h after incubation), the cells were washed thoroughly with PBS, followed by trypsinization and centrifugation at 3000 rpm for 5 min to harvest the cells as pellets. The cells were re-suspended in water, ultrasonicated and extracted by 1 mL of methanol. Finally, the amount of DOX internalized by MCF-7/ADR cells was measured by HPLC.

### In vivo studies

#### Xenograft tumor mouse model

All animal experiments were performed under a protocol approved by Henan laboratory animal center. The MCF-7/ADR tumor models were generated by subcutaneous injection of 2 × 10^6^ MCF-7/ADR cells in 0.1 mL saline into the right shoulder of nude mice (20–23 g, Shanghai Institutes for Biological Sciences, CAS). The mice were used when the tumor volume reached 60 to 100 mm^3^ (~ 10 days after tumor inoculation).

#### Pharmacokinetics and biodistribution

For pharmacokinetics: 0.2 mL of DOX (5 mg/kg), TD/DOX (DOX dosage: 5 mg/kg), DOX@liposome (DOX dosage: 5 mg/kg; liposome dosage: ~ 25 mg/kg) and LNSD (DOX dosage: 5 mg/kg; TD@liposome dosage: ~ 12 mg/kg) were intravenously injected into the tumor-free mice (3 mice per group). After injection for 0.25, 0.5, 1, 2, 4, 8, 12, and 24 h, 0.5 mL of blood was drawn from eyes of the tumor-free mice, centrifuged and then homogenized in methanol. DOX in blood samples were detected by HPLC.

For biodistribution: 0.2 mL of DOX (5 mg/kg), TD/DOX (DOX dosage: 5 mg/kg), DOX@liposome (DOX dosage: 5 mg/kg; liposome dosage: ~ 25 mg/kg) and LNSD (DOX dosage: 5 mg/kg; TD@liposome dosage: ~ 12 mg/kg) were intravenously injected into the tumor-bearing mice (3 mice per group). After injection for 24 h, the mice were killed to collect heart, liver, spleen, lung, kidney and tumor, weighed, and homogenized in buffer (methanol to saline ratio, 1:1). DOX in different tissues were determined by HPLC. Furthermore, a near-infrared dye (IR783, water-soluble) was used to mark LSND. A sample of 0.2 mL of IR783@liposome was intravenously injected into tumor-bearing mice, and the whole body fluorescence imaging was performed at 0, 0.5, 1, 6, 8, 10, 12 and 24 h after injection using a small animal imaging system (Xtreme, Bruke).

#### In vivo antitumor effect

MCF-7/ADR tumor-bearing mice were divided into 5 groups (six mice per group), minimizing the differences of weights and tumor sizes in each group. The mice were administered with (1) Saline (0.2 mL), (2) TD@liposome (0.2 mL, ~ 12 mg/kg), (3) DOX (5 mg/kg, 0.2 mL), (4) DOX@liposome (DOX: 5 mg/kg; liposome: ~ 25 mg/kg), (5) LNSD (DOX: 5 mg/kg; TD@liposome: ~ 12 mg/kg, 0.2 mL) were intravenously injected into mice via the tail vein every 2 days, respectively. The mice were observed daily for clinical symptoms and the tumor sizes were measured by a caliper every other day and calculated as the volume = (tumor length) × (tumor width)^2^/2. After treatment for 15 days, the mice were sacrificed to collect tumor for H&E and TUNEL staining.

### Statistical analysis

Quantitative data are expressed as mean ± SD and analyzed by use of Student’s t test. P values < 0.05 were considered statistically significant.

## Availability of data and materials

All data generated or analyzed during this study are included in this published article.

## Supplementary information

**Additional file 1.** Size distributions of nanopreparations.

**Additional file 2.** Encapsulation efficiency of DOX in nanopreparations.

**Additional file 3.** Photos of DOX@liposome and LNSD in PBS.

**Additional file 4.** Cellular distribution of FAM-TD@liposome.

**Additional file 5.** Co-localization ratio of DOX and TD in cells.

**Additional file 6.** Cell viability of MCF-7/ADR cells treated with different concentrations of blank carrier.
