# Supplementary material for: Ultrasmall nanostructured drug based pH-sensitive liposome for effective treatment of drug-resistant tumor
Source: J Nanobiotechnology. 2019 Nov 29;17:117. doi: 10.1186/s12951-019-0550-7 (PMC6884872; doi:10.1186/s12951-019-0550-7)
Supplement: Supplementary file 1 — Additional file 1. Size distributions of nanopreparations. [file 12951_2019_550_MOESM1_ESM.docx]

**Additional file**

**Ultrasmall nanostructured drug based pH-sensitive liposome for effective treatment of drug-resistant tumor**

Yanyan Li^1^, Yongxia Zhai^1^, Wei Liu^2^, Kaixiang Zhang^2,3,4*^, Junjie Liu^2,3,4*^, Jinjin Shi^2,3,4*^ and Zhenzhong Zhang^2,3,4^

^1^ Department of Pharmacy, the Fifth Affiliated Hospital of Zhengzhou University, Kangfu Road, Zhengzhou 450052, China

^2^School of Pharmaceutical Sciences, Zhengzhou University, Zhengzhou, PR China.

^3^Collaborative Innovation Center of New Drug Research and Safety Evaluation, Henan Province, PR China.

^4^Key laboratory of Targeting Therapy and Diagnosis for Critical Diseases, Henan Province, PR China.

*Correspondent author: Dr. Kaixiang Zhang, Dr. Junjie Liu and Dr. Jinjin Shi

Tel: 86-371-67781910

Fax: 86-371-67781908

E-mail:zhangkx@zzu.edu.cn, [liujunjie@zzu.edu.cn](mailto:liujunjie@zzu.edu.cn) and shijinyxy@zzu.edu.cn

**
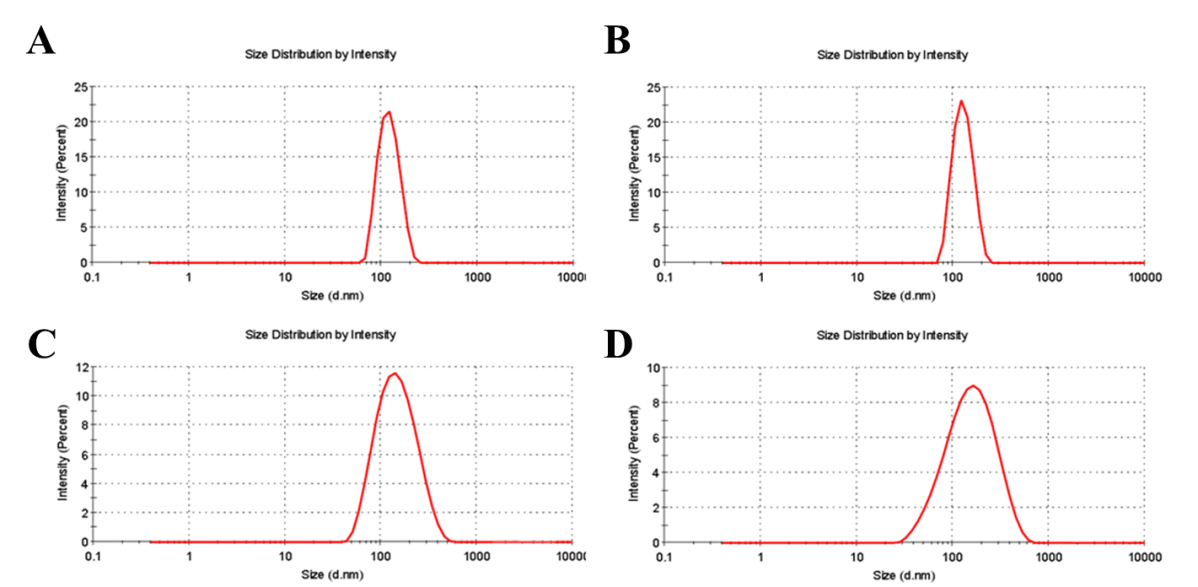
**

**Additional file 1. Size distributions of liposome (A), DOX@liposome (B), TD@liposome (C) and LNSD (D).**
