# Supplementary material for: Ultrasmall nanostructured drug based pH-sensitive liposome for effective treatment of drug-resistant tumor
Source: J Nanobiotechnology. 2019 Nov 29;17:117. doi: 10.1186/s12951-019-0550-7 (PMC6884872; doi:10.1186/s12951-019-0550-7)
Supplement: Supplementary file 2 — Additional file 2. Encapsulation efficiency of DOX in nanopreparations. [file 12951_2019_550_MOESM2_ESM.docx]

**
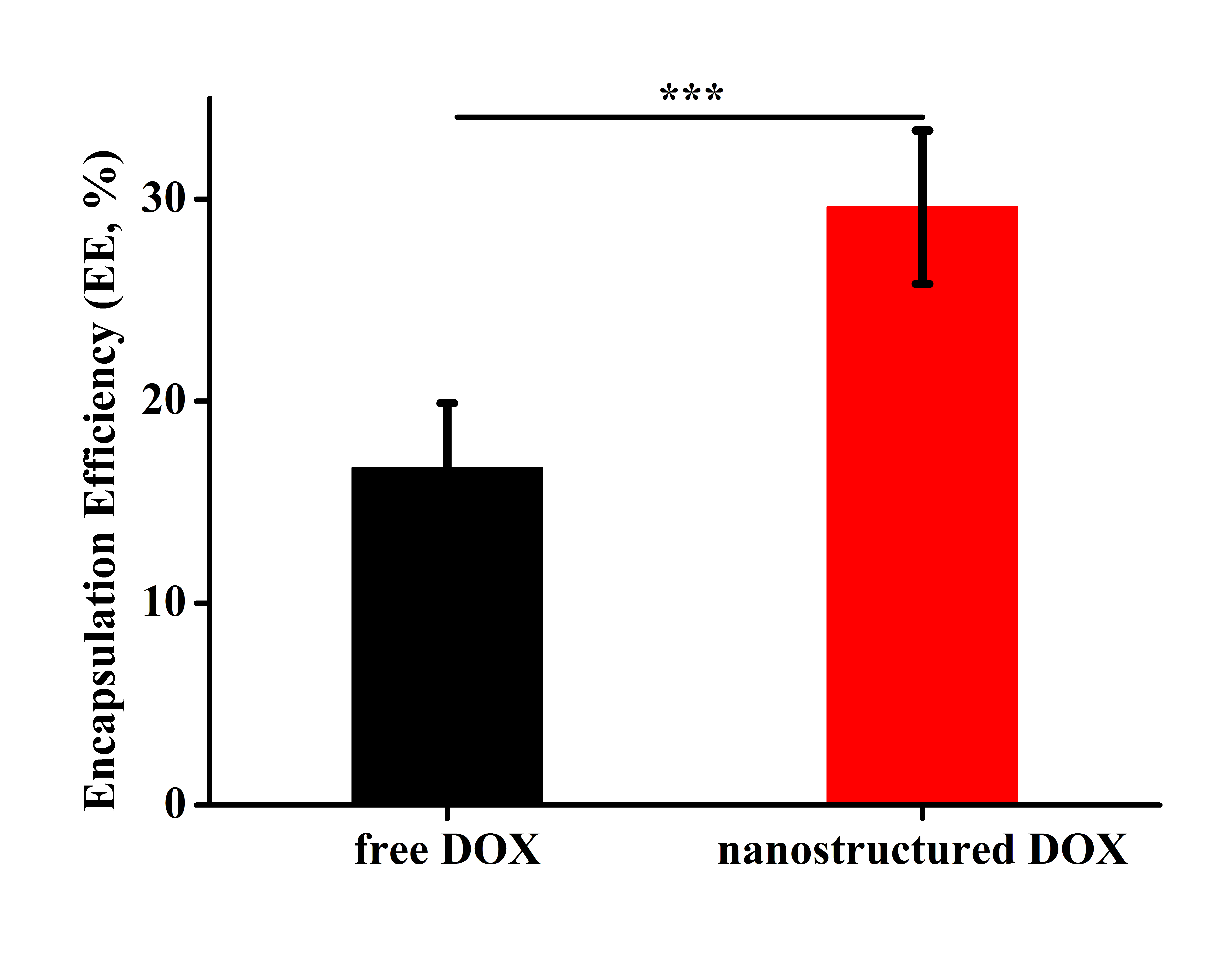
**

**Additional file 2. Encapsulation efficiency of DOX@liposome (free DOX strategy) and LNSD (nanostructured DOX strategy)** (n=6). Data presented are means ± SD. ***, *p*<0.01.
