# Supplementary material for: Ultrasmall nanostructured drug based pH-sensitive liposome for effective treatment of drug-resistant tumor
Source: J Nanobiotechnology. 2019 Nov 29;17:117. doi: 10.1186/s12951-019-0550-7 (PMC6884872; doi:10.1186/s12951-019-0550-7)
Supplement: Supplementary file 3 — Additional file 3. Photos of DOX@liposome and LNSD in PBS. [file 12951_2019_550_MOESM3_ESM.docx]

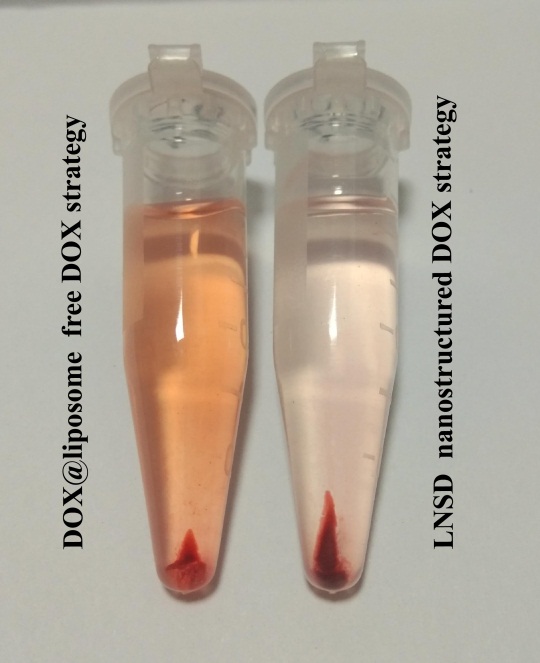


**Additional file 3.** **Photos of DOX@liposome and LNSD in PBS (pH 7.4) for 12 h after centrifugation**, indicating LNSD could significantly decrease the leakage of DOX.
