# Supplementary figures and images for: Ultrasmall nanostructured drug based pH-sensitive liposome for effective treatment of drug-resistant tumor
Source: J Nanobiotechnology. 2019 Nov 29;17:117. doi: 10.1186/s12951-019-0550-7 (PMC6884872; doi:10.1186/s12951-019-0550-7)

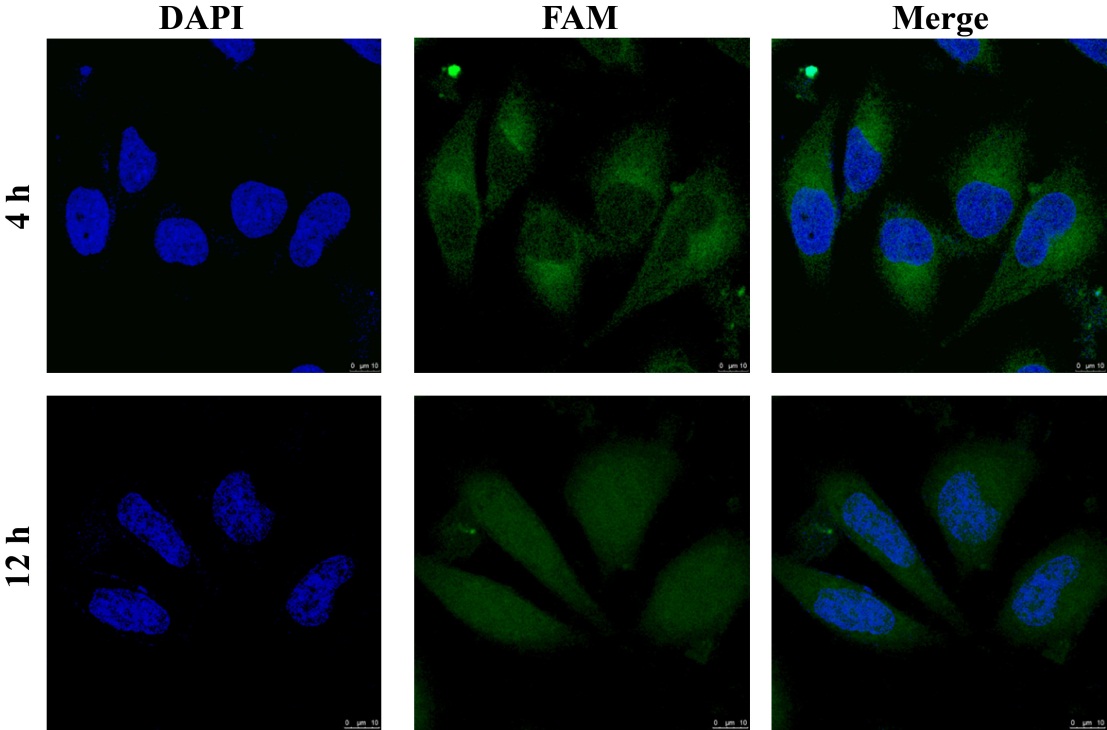


**Additional file 4.** **Cellular distribution of FAM-TD@liposome in MCF-7/ADR cells for 4 and 12 h.** Scale bar: 10 μm.

Supplement: Supplementary file 4 — Additional file 4. Cellular distribution of FAM-TD@liposome. [file 12951_2019_550_MOESM4_ESM.docx]
