# Supplementary material for: Ultrasmall nanostructured drug based pH-sensitive liposome for effective treatment of drug-resistant tumor
Source: J Nanobiotechnology. 2019 Nov 29;17:117. doi: 10.1186/s12951-019-0550-7 (PMC6884872; doi:10.1186/s12951-019-0550-7)
Supplement: Supplementary file 5 — Additional file 5. Co-localization ratio of DOX and TD in cells. [file 12951_2019_550_MOESM5_ESM.docx]

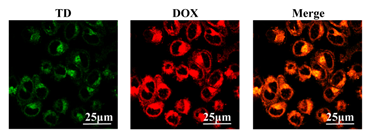


**Additional file 5.** **Representative CLSM images of cells treated with DOX/TD@liposome for 4h.** Scale bar: 25 μm.
