# Supplementary material for: Ultrasmall nanostructured drug based pH-sensitive liposome for effective treatment of drug-resistant tumor
Source: J Nanobiotechnology. 2019 Nov 29;17:117. doi: 10.1186/s12951-019-0550-7 (PMC6884872; doi:10.1186/s12951-019-0550-7)
Supplement: Supplementary file 6 — Additional file 6. Cell viability of MCF-7/ADR cells treated with different concentrations of blank carrier. [file 12951_2019_550_MOESM6_ESM.docx]

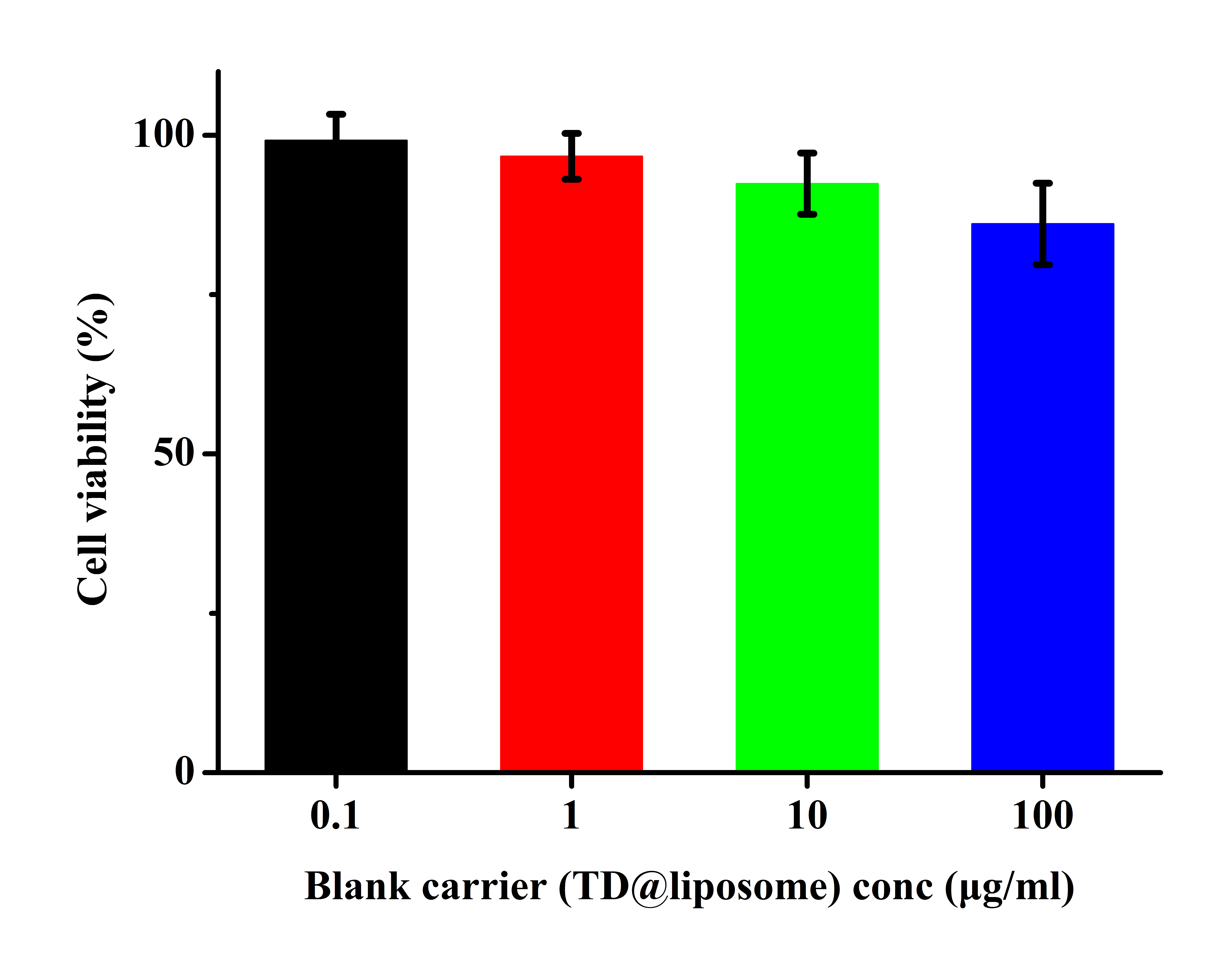


**Additional file 6.** **Cell viability of MCF-7/ADR cells treated with different concentrations of blank carrier** (TD@liposome, n=6).
